# Combination of Auranofin and ICG-001 Suppress the Proliferation and Metastasis of Colon Cancer

**DOI:** 10.3389/fonc.2021.738085

**Published:** 2021-11-24

**Authors:** Zhaoyan Lin, Qingqing Li, Ying Zhao, Zixiang Lin, Nan Cheng, Di Zhang, Gang Liu, Jiahao Lin, Hong Zhang, Degui Lin

**Affiliations:** ^1^ Department of Veterinary Clinical Sciences, College of Veterinary Medicine, China Agricultural University, Beijing, China; ^2^ College of Animal Science and Technology, Hainan University, Haikou, China

**Keywords:** ICG-001, auranofin, colon cancer, proliferation, metastasis

## Abstract

Colon cancer is one of the deadliest tumors in the world, and with high metastasis rate and mortality, effective drugs for its treatment are still in need. Auranofin (AF) is a gold complex that has been attested by FDA for treating human rheumatism, and researchers have found that AF acts as a great antitumor drug in recent years. ICG-001 is a small molecule inhibitor of Wnt/β-catenin pathway. In the present study, we aimed to explore the synergistic antitumor effects and the underlying mechanisms of AF and ICG-001 combination therapy on human colon cancer. The results showed that AF and ICG-001 synergistically depressed the growth and invasion of human colon cancer cells by inhibiting the phosphorylation of Signal Transducer and Activator of Transcription 3 (STAT3) and its downstream mediator B-cell lymphoma-2-like 1 (Bcl-xL) and inducing caspase-3-dependent apoptosis. Moreover, AF combined with ICG-001 synergistically inhibited the growth of colon cancer in subcutaneous xenograft mice models and restrained metastasis in lung metastasis mice models. In conclusion, our results demonstrated that combination of AF and ICG-001 suppressed the proliferation and metastasis of colon cancer by inhibiting STAT3 phosphorylation. Therefore, this combination therapy may possess potential therapeutic properties for human colon cancer.

## Introduction

Colon cancer is one of the deadliest tumor in the world, with high metastasis rate and mortality (1). Although surgery is the most common treatment for patients with early-stage colon cancer, chemotherapy and target drugs are still necessary for those with advanced-stage and metastasis cancer. Moreover, according to the statistics of United States, the 5-year survival rates for patients with colon cancer from 2001~2003 to 2004~2009 increase only by 0.9% (from 63.7 to 64.6%) (2), and the 5-year relative survival rate for those patients is 64% until year 2019 (3), indicating that the novel therapy for colon cancer is still under urgent need.

Combination therapy is using multiple drugs for treating diseases and reducing suffering. The possible favorable outcomes for synergism include (1) increasing the drug efficacy; (2) decreasing the dosage but increasing or maintaining the same efficacy to avoid toxicity; (3) minimizing or slowing down the development of drug resistance; and (4) providing selective synergism against target (or efficacy synergism) *versus* host (or toxicity antagonism) (4). Therefore, drug combinations have been widely used and become the leading choice for treating the most dreadful diseases, such as cancer and infectious diseases (4).

Auranofin (AF) is a gold complex that has been attested by FDA for treating human rheumatism. Researchers have found that AF acts as a great antitumor antigen in recent years, for example, colon cancer (5, 6), non-small-cell lung cancer (7), prostate cancer (8), breast cancer (9), and ovarian cancer (10). Besides, AF also has a great potential in combination with other drugs (11–13).

ICG-001 is a small molecule inhibitor of Wnt/β-catenin pathway. The Wnt/β-catenin pathway normally acts as a critical part in initiation and proliferation in multiple tumors, including colon cancer (14–17). To develop this capability, β-catenin recruits cyclic AMP response element-binding protein (CBP) to generate a transcriptionally active complex (16). ICG-001 acts as a Wnt/β-catenin inhibitor by specially interacting with CBP and competing with β-catenin for CBP (17). Recent researches have found antitumor effect of ICG-001 for multiple cancer types, such as colon cancer (18), pancreatic cancer (19), gastric cancer (20), and uveal melanoma (21), by leading cell cycle arrest or apoptosis. Meanwhile, ICG-001 also performed well with other drugs to obtain synergistic antitumor effect (15, 22).

STAT3 is a key signaling protein engaged by a multitude of growth factors and cytokines to elicit diverse biological outcomes including cellular growth, differentiation, and survival (23, 24). Persistent activation of STAT3 signaling is frequently detected in human colon cancers (25, 26), and in association with invasion, survival, and growth of colorectal cancer cells (26–29). STAT3 phosphorylation at tyrosine 705 (Tyr 705) leads to dimerization, nuclear translocation, recognition of STAT3-specific DNA binding elements, and upregulation of various downstream target genes, such as B-cell lymphoma-2-like 1 (Bcl-xL), B-cell lymphoma-2 (Bcl-2), myeloid cell leukemia sequence 1 (Mcl1), survivin, c-Myc, cyclin D1, and others (24, 30–32).

Since STAT3 is a potential drug target for colon cancer therapy (32), therefore, the purpose of our study was to explore the combination effect of AF and ICG-001 on colon cancer and the underling mechanism related to STAT3. In the present study, we found that AF and ICG-001 synergistically depressed the growth and metastasis of human colon cancer *in vitro* and *in vivo*. Further, we also indicated that this potential drug combination directly inhibited the phosphorylation of STAT3 (p-STAT3).

## Methods and Materials

### Cell Culture

The human colon cancer cell line HCT-116 (Cell bank of the Chinese Academy of Science, Beijing, China) was cultured in McCoy’s 5A (Life Technologies Inc., Carlsbad, CA, USA). The human colon cancer cell lines SW-480 and HCT-116 with luciferase tdT (HCT116-Luc-tdT) (Cell bank of the Chinese Academy of Science, Beijing, China) were cultured in Iscove’s Modified Dulbecco’s Medium (IMDM, Life Technologies Inc., Carlsbad, CA, USA). The human colon cancer cell line DLD-1 (Cell bank of the Chinese Academy of Science, Beijing, China) was cultured in Dulbecco’s Modified Eagle Medium (DMEM, Life Technologies Inc., Carlsbad, CA, USA). All mediums supplemented with 10% fetal bovine serum (FBS, Life Technologies Inc., CA USA), penicillin (100 units/ml), and streptomycin (100 mg/ml) (Life Technologies Inc., CA, USA). All cells were cultured at 5% CO_2_, 37°C in humidifier incubator.

### Drug Treatment

AF was purchased from MedChemExpress Inc. (CAS No.: 34031-32-8, Monmouth Junction, NJ, USA); ICG-001 was purchased from MedChemExpress Inc. (CAS No.: 780757-88-2, Monmouth Junction, NJ, USA); Interleukin-6 (IL-6) was purchased from Life Technologies Inc. (PHC0063, Carlsbad, CA, USA). For the cell co-treatment, the cells were incubated with AF and ICG-001 premixed under the dose ratio of 1:10 for 24 h; for the experiments regarding the IL-6 activation, the cells were post-treated with 50 ng/ml IL-6 dissolved in culture medium for 15 min after the drug treatment; for the mice co-treatment, mice were treated with AF and ICG-001 premixed under the dose ratio of 1:2.

### 3D Cell Culture Model

A 96-well cell culture plate was coated with Matrigel (BD Biosciences, MA, USA), and 1×10^4^ cells resuspended with drugs (2 μM AF, 20 μM ICG-001, 2 μM AF + 20 μM ICG-001) in culture medium were seeded into the plate and cultured in 37°C for 30 min. Then the culture mediums with 10% Matrigel were added into the plate and cultured for 4 days (33, 34). Then the diameters of the cell spheroid were measured, and spheroids were stained with Calcein-AM (CA1630, Solarbio, Beijing, China) to detect the living cells. The images were taken by microscope camera system (CKX41, OLYMPUS, Monolith, Japan).

### Cell Viability Assay

CCK-8 assay (TransGen Biotech, Beijing, China) was used to measure cell viability. Briefly, 1,000 cells/well were co-incubated with different doses of AF, ICG-001, and the combination of both drugs in a 96-well cell culture plates for 24 h. Later, the value of OD_450nm_ of each well was obtained and recorded with a microplate reader by using CCK-8 assay. Cell viability was calculated based on surviving cell number (%treated/untreated); surviving cell number of control group corresponded to 100%. The experiments were carried out in triplicates, and the half inhibition rate (IC_50_) values were calculated. The combination index (CI) was also calculated to examine the interaction between AF and ICG-001 by Compusyn software (version 1.0, Inc., Paramus, NJ, 07652 USA). CI values 1, <1, and >1 indicated an additive effect, synergism, or antagonism, respectively.

### Cell Migration Assay

A scratch and wound healing assay was performed to measure cell migration. Cells (3 × 10^5^ cells/well) were plated into six-well cell culture plates. Cells were grown to confluence and were scratched crossly with sterile 1,000 μl pipette tips. After scratching, cells were washed twice and co-incubated with drugs (2 μM AF, 20 μM ICG-001, 2 μM AF + 20 μM ICG-001) for 24 h. The width of wound in each well was recorded by a microscope camera system (CKX41, OLYMPUS, Monolith, Japan), and the change rate of the wound width of each well was calculated.

### Cell Invasion Assay

Twenty-four-well inserts (Costar, New York, NY, USA) coated with Matrigel were used for cell invasion assays. Briefly, cells were diluted into 1 × 10^4^ cells/ml, resuspended in 100 μl serum-free medium with drugs (1 μM AF, 10 μM ICG-001, 1 μM AF + 10 μM ICG-001), and seeded into the upper chamber, while the lower chamber was filled with the medium with 10% FBS. Cells attaching to the bottom side of the upper chamber were stained with 0.1% crystal violet (Solarbio, Beijing, China) and counted under microscope 24 h later.

### Colony Formation Assay

Cells were plated 1,000 cells/well in 6-well plates with drugs (1 μM AF, 10 μM ICG-001, 1 μM AF + 10 μM ICG-001) and further cultured at 37°C, 5% CO_2_ for 10 days. Colony formation analysis was done by staining with 0.1% crystal violet (Solarbio, Beijing, China), and cell proliferating states were recorded by photography and counted manually.

### Western Blot Assay

Protein was extracted from harvested cells or tumor and lung tissues from mice and quantified through bicinchoninic acid (BCA) analysis (Life Technologies Inc., CA, USA). Equal amounts of protein were loaded. The following primary antibodies were used for Western blot analysis: Phospho-Stat3 (Tyr705) (D3A7) (9145T, Cell Signaling Technology, Danvers, MA, USA, 1:2,000), Stat3 (9139T, Cell Signaling Technology, Danvers, MA, USA, 1:2,000), Bcl-xL (54H6) (2764T, Cell Signaling Technology, Danvers, MA, USA, 1:2,000), Caspase-3 (9662S, Cell Signaling Technology, Danvers, MA, USA, 1:1,000), cleaved-Caspase-3 (9661T, Cell Signaling Technology, Danvers, MA, USA, 1:1,000),α-Tubulin (66031-1-Ig, Proteintech, Rosemont, IL, USA, 1:2,000), and GAPDH (60004-1-lg, Proteintech, Rosemont, IL, USA, 1:2,000). After incubating with primary antibodies and washed five times, the membranes were incubated with secondary antibodies conjugated with horseradish peroxidase (HRP) anti-mouse/rabbit IgG (SA00001–9, SA00001–1, Proteintech, Rosemont, IL, USA, 1:5,000), washed five times, and exposed under chemiluminescent imaging analysis system (Tanon 5200, China). Densitometry analysis was done by Image J (Version 1.50i, National Institutes of Health, USA).

### Transient Transfection of STAT3 siRNA

Commercial STAT3 siRNA was obtained from Oligobio Inc. (Beijing, China) and used to target human STAT3 (Gene ID: 6774). Cells were transfected with siRNA (50 nM) for 24 h before drug treatment by using Lipofectamine™ 2000 Transfection Reagent (11668019, Life Technologies Inc., CA USA) according to the manufacturer’s instructions. Non-specific siRNA (NC siRNA, Oligobio Inc., Beijing, China) was used as a negative control. A western blot assay was done to confirm the selective silencing of STAT3 and then detect the expression of Bcl-xL, Caspase-3 and cleaved-Caspase-3 of STAT3-slienced cells treated with drugs compared with those of control cells.

### Immunofluorescence Assay

Cells (5 × 10^4^ cells/ml) were plated on coverslips, treated with drugs (2 μM AF, 20 μM ICG-001, 2 μM AF + 20 μM ICG-001) for 24 h, fixed in 4% paraformaldehyde solution for 20 min at room temperature, and permeabilized in 0.5% Triton X-100 for 5 min. Then, the cells were washed twice, incubated with anti-Phospho-Stat3 antibodies for overnight at 4°C, then incubated with appropriate conjugated secondary antibodies for 1 h at 37°C. Then the coverslips were mounted on slides with DAPI (S2110, Solarbio, Beijing, China). The slides were visualized using a Nikon Confocal microscope, and pictures were taken with NIS-Element Viewer (version 5.21.0, Nikon Instruments Inc., Shanghai, China).

### TUNEL Assay

Deoxynucleotidyl Transferase-Mediated dUTP Nick End Labeling (TUNEL) assay was performed to measure cell apoptosis. Briefly, 5×10^4^ cells were plated on cell culture slides in 12-well plates. When cells were approximately 70% confluent, they were then treated with drugs (2 μM AF, 20 μM ICG-001, 2 μM AF + 20 μM ICG-001) for 24 h. After fixation in 4% paraformaldehyde for 1 h, samples were stained with TUNEL reagent (TransGen, Beijing, China) following the manufacturer’s instructions. Then images were captured with Nikon confocal microscope.

### Tumor Xenograft Mouse Model

The animal study was reviewed and approved by the Animal Ethics Committee of the China Agricultural University (approval code AW12601202-2-1), according to the guidelines for Laboratory Animal Use and Care from the Chinese Center for Disease Control and Prevention and the Rules for Medical Laboratory Animals (1998) from the Chinese Ministry of Health, under protocol CAU20151001-1.

To establish a tumor xenograft mouse model, 1 × 10^6^ cells of HCT-116 were resuspended in 150 μl PBS and injected subcutaneously into 5-week-old male null-balb/c mice (Vital River, Beijing, China). Tumor sizes were measured with a digital caliper every 3 days. The volume of tumor size was calculated as follows: V = L×W^2^/2, of which *W* corresponded to the width and *L* to the length. When the length reached about 5 mm, mice were then randomly assigned into four groups and treated with drugs (AF 5 mg/kg, ICG-001 10 mg/kg, AF 5 mg/kg + ICG-001 10 mg/kg) or vehicle by intraperitoneal injection daily for another 14 days. At the end of treatment, all mice were sacrificed and xenograft tumors were harvested. To evaluate the drug toxicity, (1) mice body weight was measured every day during treatment; (2) the blood biochemistry indexes of mice were detected; (3) the lungs, livers, kidneys, spleens, and hearts were harvested to perform pathological section with HE staining at the end of treatment.

### Pulmonary Metastasis Mouse Model

To establish a pulmonary metastasis mouse model, 1 × 10^6^ cells of HCT116-Luc-tdT were injected into the tail vein of 5-week-old male null-balb/c mice (Vital River, Beijing, China). On the second day after injection, the mice were randomly assigned into four groups and treated with drugs (AF 5 mg/kg, ICG-001 10 mg/kg, AF 5 mg/kg + ICG-001 10 mg/kg) or vehicle by intraperitoneal injection daily for 14 days. The fluorescence intensity of living mouse lung and lungs collected from sacrificed mice at the end of treatment was measured by using *in vivo* imaging system (IVIS Spectrum, PerkinElmer Inc., USA). The lung tissues were also harvested to perform a pathological section with HE staining and to measure the metastatic area.

### Immunohistochemistry

Xenograft tumor tissues collected from xenograft nude mice and lung tissues collected from the pulmonary metastasis mice were fixated with 10% (v/v) neutral-buffered formalin (Biosharp, Beijing, China). Then samples were embedded in paraffin wax and sectioned into 3 μm slides. After deparaffination and antigen retrieval with Sodium Citrate-Hydrochloric acid Buffer solution, sections were incubated with primary antibodies: Phospho-Stat3, Bcl-xL, and cleaved-Caspase-3 at 4°C overnight, followed by incubation of biotinylated secondary antibodies at 37°C for 1 h. Sections were stained with diaminobenzidine (Solarbio, Beijing, China) and counterstained with hematoxylin (ZSGB-BIO, Beijing, China). Images were captured with a bright field digital microscope.

### Statistical Analysis

Statistical analysis was performed by using GraphPad Prism5 software (version 5, GraphPad Software Inc., San Diego, CA, USA). A two-tailed unpaired t-test with Welch’s correction was applied when the variances of two groups were proved equal by the F test, and *p* values of 0.05 or less were the threshold for statistical significance.

## Results

### AF and ICG-001 Synergistically Inhibits the Proliferation of Colon Cancer *In Vitro*

**Figure 1:**
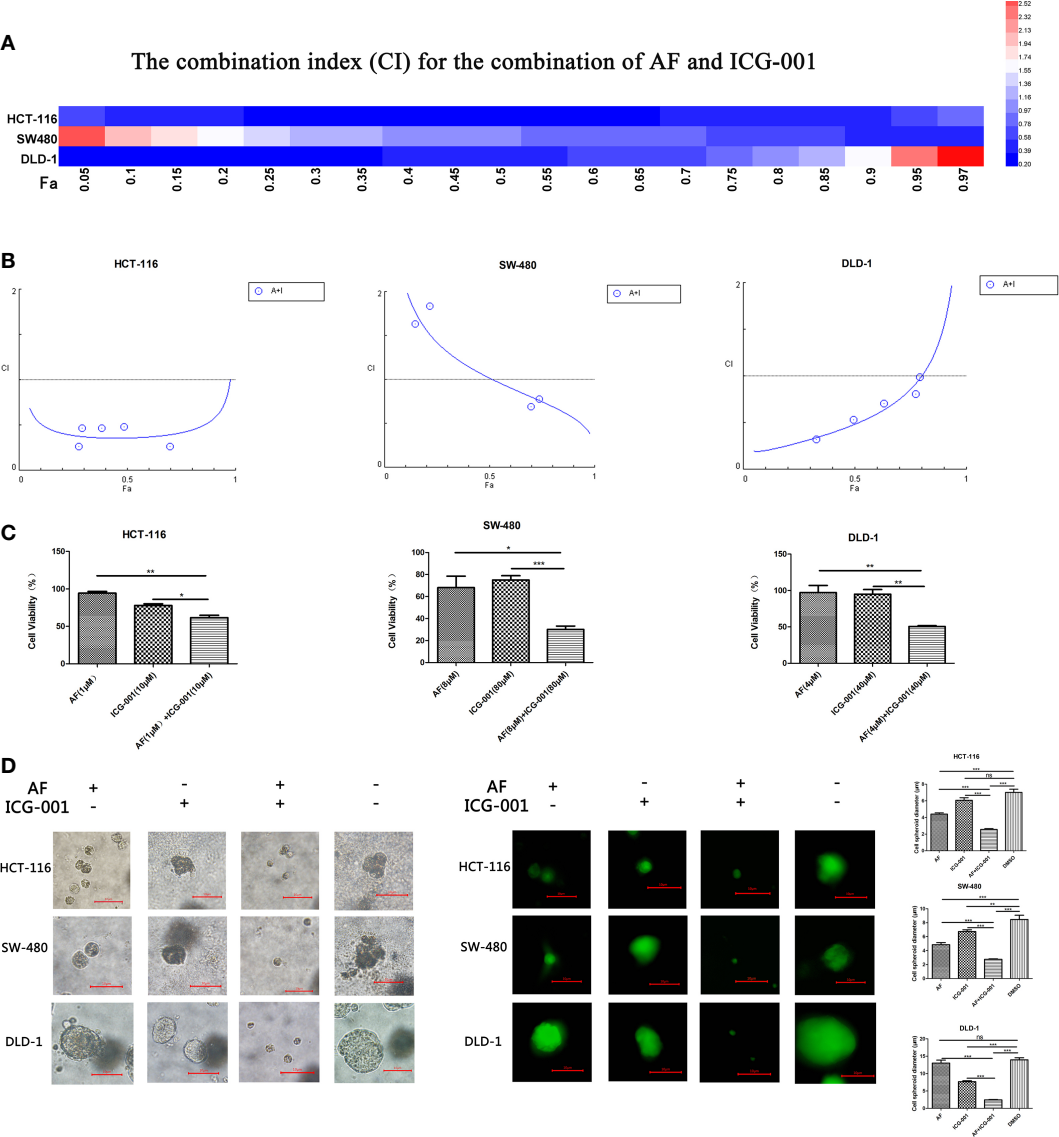
AF and ICG-001 synergistically suppressed the proliferation of colon cancer *in vitro*. **(A, B)** The combination index (CI) for the combination therapy could reach <1 in all three cell lines, indicating the combination of AF and ICG-001 could synergistically inhibit the growth of colon cancer *in vitro*. Fa, fraction affected. **(C)** The cell viability of colon cancer cell lines after application of drugs combination or alone for 24 h; the number of surviving cells in control group corresponds to 100%. **(D)** AF and ICG-001 synergistically inhibit the growth of 3D cell spheroids of three colon cancer cell lines. Bar=10 μm. Data were representative of three independent experiments. *p*-values: *p < 0.05; **p < 0.01; ***p < 0.001. ns, non-significant.

According to the cell viability assay, AF and ICG-001 both suppressed the proliferation of colon cancer cell lines. The IC_50_ of AF in HCT-116, SW-480, DLD-1 cell lines were 7.85, 9.68, 7.74 μM, respectively. The IC_50_ of ICG-001 in HCT-116, SW-480, DLD-1 cell lines were 106.39, 264.66, 2,697.72 μM, respectively (**Supplementary Table S1**). In addition, the CI value was calculated to describe the combination effect of drugs, and the CI value could reach <1 in all three cell lines (**Figures 1A, B**). For example, the CI value of HCT-116, SW-480, DLD-1 at 70% cell growth inhibited was 0.41769, 0.74747, and 0.86046, respectively (**Supplementary Table S1**). Those results indicated the combination of these two drugs might synergistically inhibit the growth of colon cancer *in vitro*. Then we confirmed that combination application of AF and ICG-001 could significantly produce a more marked inhibitory effect on three colon cancer cell lines than treatment with AF or ICG-001 alone (**Figure 1C**).

The 3D cell culture models can be used to culture cancer stem cell for drug screening, and provide more *in vivo*–like results than *in vitro* (35). So, we arranged a 3D cell culture assay and detected the diameter of the cell spheroid after drug treatment (**Figure 1D**). Moreover, the living cells were stained with Calcein-AM to confirm the results (**Figure 1D**). Those results indicated that combination of AF and ICG-001 could synergistically inhibit the growth of cell spheroid.

### Combination of AF and ICG-001 Suppresses Migration, Colony Formation, and Invasion of Colon Cancer *In Vitro*

**Figure 2:**
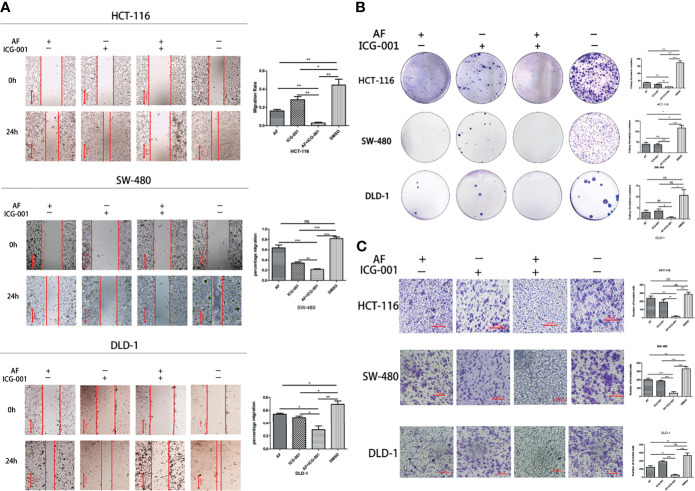
Combination of AF and ICG-001 Suppressed Migration **(A)**, Colony Formation **(B)**, and Invasion **(C)** of Colon Cancer *in vitro*. Bar = 200 μm **(A)** and 100 μm **(C)**. Data were representative of three independent experiments. *p*-values: *p < 0.05; **p < 0.01; ***p < 0.001. ns, non-significant.

To assess whether the combination therapy would suppress cell migration ability, clonogenic activity, and invasion ability, we performed the cell migration assay, colony forming assay, and cell invasion assay, respectively. Our results showed that the combination use of AF and ICG-001 could significantly inhibit cell migration (**Figure 2A**), colony formation (**Figure 2B**), and invasion (**Figure 2C**) of colon cancer cells compared with those in the control group or the individual groups.

### Combination of AF and ICG-001 Induces Apoptosis by Downregulating the Phosphorylation of STAT3 *In Vitro*

**Figure 3:**
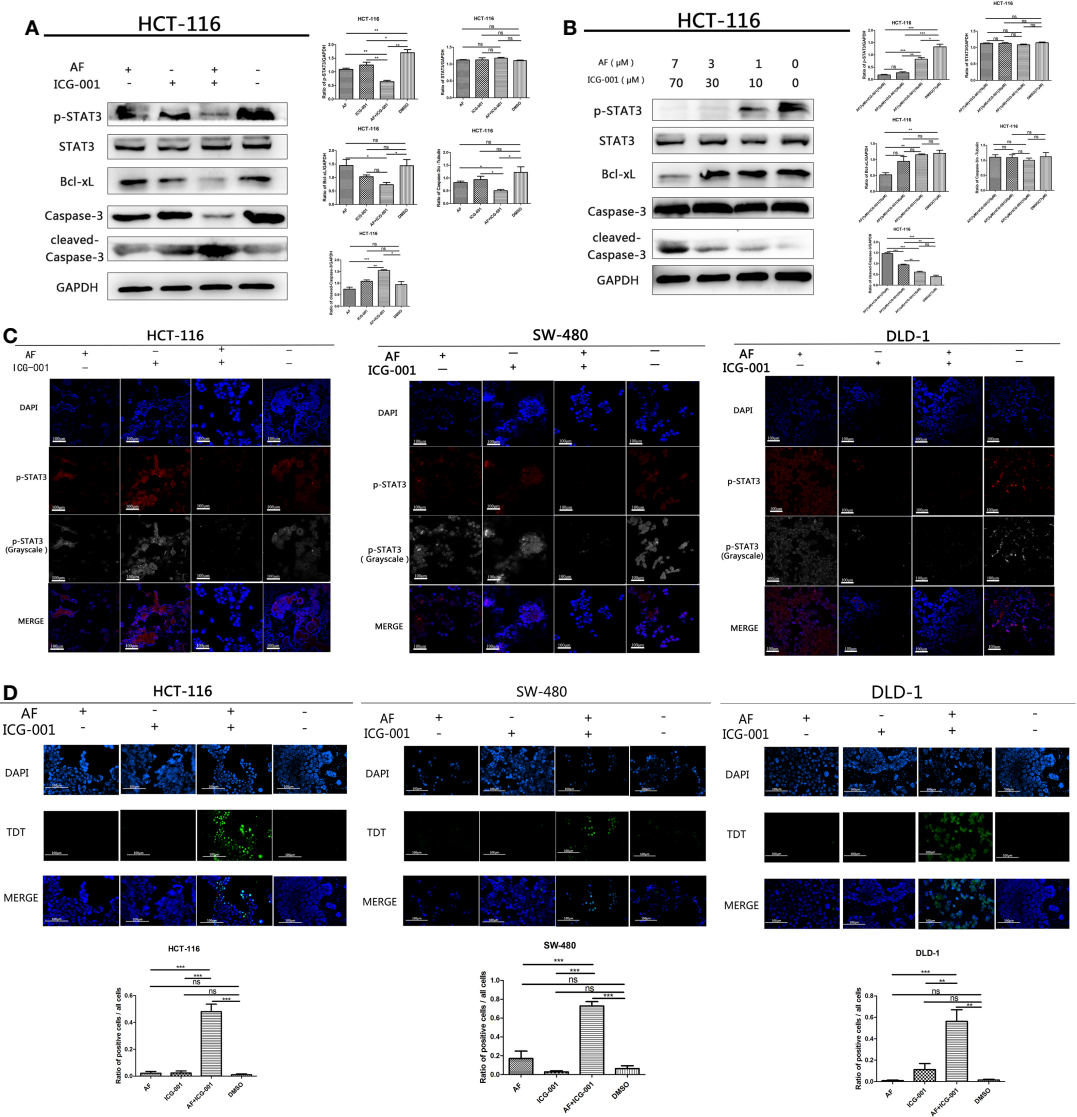
AF and ICG-001 displayed synergistic effect by downregulating the phosphorylation of STAT-3, and Bcl-xL, inducing apoptosis on colon cancer cell lines. **(A)** Western Blot of HCT-116 cell line treated with AF (7 μM), ICG-001 (70 μM), the combination of both drugs, and DMSO as control for 24h **(B)** Western Blot of HCT-116 cell line treated with the combination of AF and ICG-001 in different concentration, and DMSO as control for 24h **(C)** The immunofluorescence assay of HCT-116, SW-480, and DLD-1 cell lines. Bar = 100 μm. **(D)** TUNEL assay of HCT-116, SW-480, DLD-1 cells treated with drugs, and the green signals indicated apoptotic cells (excitation wavelength: 488 nm). Bar = 100 μm. Data were representative of three independent experiments. *p*-values: *p < 0.05; **p < 0.01; ***p < 0.001. ns, non-significant.

The high-level p-STAT3 expression is frequently reported in colon cancer (26), so we suppose that the combination application of AF and ICG-001 may involve in regulation of STAT3-related pathways. And our results indicated that AF or ICG-001 alone could significantly suppress the expression of p-STAT3 than that in the control group, but the combination therapy exerted a greater inhibitory effect on p-STAT3 expression than the individual drug treatment (**Figure 3A**). In addition, the combination treatment significantly downregulated the expression of p-STAT3 with a concentration-dependent manner (**Figure 3B**). Then, this differential expression of p-STAT3 among different groups was confirmed by using immunofluorescence assay (**Figure 3C**). Furthermore, the expression of Bcl-xL, which is a downstream mediator of p-STAT3, also was significantly inhibited with a concentration-dependent manner in the combination group compared with that in other groups, while the expression level of cleaved-caspase 3 was significantly upregulated with a concentration-dependent manner in the combination group compared with that in other groups (**Figures 3A, B**). To confirm the apoptosis induced by the combination of AF and ICG-001, a TUNEL assay was utilized. As the results showed, the number of apoptotic cells was significantly higher in the combination group than those in AF or ICG-001 alone or the control group (**Figure 3D**).

**Figure 4:**
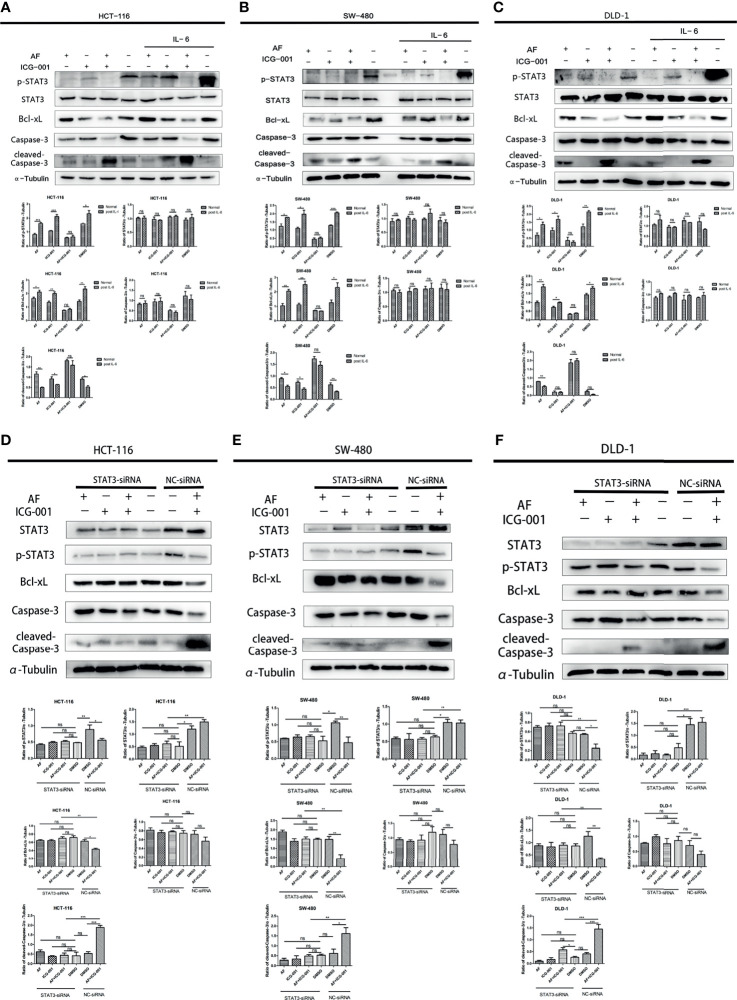
Western Blot analysis of colon cancer cell lines. HCT-116 **(A)**, SW-480 **(B)**, and DLD-1 **(C)** cells were treated with AF (7 μM), ICG-001 (70 μM), the combination of both drugs, and DMSO as control for 24 h, and for the IL-6 treatment groups, the medium was changed into 50 ng/ml IL-6 dissolved in culture medium for 15 min after drug treatment. HCT-116 **(D)**, SW-480 **(E)**, and DLD-1 **(F)** cells were transfected with STAT3-siRNA (50 nM) for 24 h, then treated with AF (7 μM), ICG-001 (70 μM), the combination of both drugs, and DMSO as control for 24h NC, negative control. Data were representative of three independent experiments. *p*-values: *p < 0.05; **p < 0.01; ***p < 0.001. ns, non-significant.

IL-6 can be an activator to induce the phosphorylation of STAT3, so in order to further explore the antitumor mechanism, we treated cancer cells with IL-6 after the combination therapy. The results showed that the p-STAT3 level was significantly regressed in the control group, AF alone group, and ICG-001 alone group, while the suppression of p-STAT3 in the combination group could not be rescued by IL-6 (**Figures 4A–C**). Simultaneously, the suppression of Bcl-xL and the promotion of cleaved-Caspase-3 could not also be rescued by IL-6 in the combination group (**Figures 4A–C**). Those indicated that the antitumor mechanism of the AF and ICG-001 combination may be through directly decreasing the level of p-STAT3. To confirm it, STAT3 were knocked down in CRC cell lines by siRNA silencing, and the western blot assay results showed this siRNA could effectively reduce the expression of STAT3 and p-STAT3 compared with the negative control groups (**Figures 4D–F**). Then the results showed the combination drug treatment could not induce the upregulation of cleaved-Caspase-3 and downregulation of Bcl-xL in STAT3 knockdown cells (**Figures 4D–F**). These indicated that the synergistical antitumor effect of the AF and ICG-001 combination could be exerted by inducing caspase-3-dependent apoptosis *via* directly inhibiting phosphorylation of STAT3 of colon cancer cells.

### Combination of AF and ICG-001 Retrains the Tumor Growth *In Vivo*

**Figure 5:**
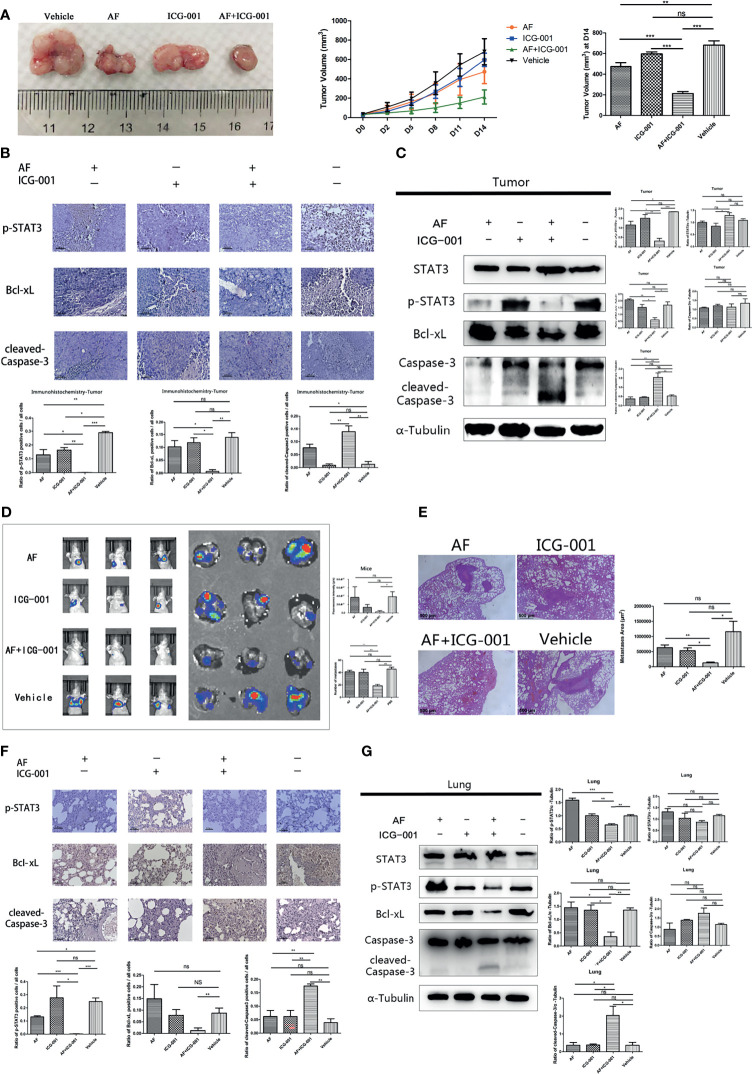
Combination of AF and ICG-001 inhibited tumor growth and metastasis *in vivo*. **(A)** The tumors were harvested by the end of the treatment; the tumor volume was measured every 3 days. AF, n = 11; ICG-001, n = 12; AF + ICG-001, n = 14; Vehicle, n = 10. **(B)** The expression of p-STAT3, Bcl-xL, and cleaved-Caspase-3 were detected by immunohistochemistry in the xenograft tumor tissues, Bar = 100 μm. **(C)** Western Blot assay performed on the tumor tissue harvested from the xenograft tumor model mice. **(D)** *In vivo* imaging system were used to measure the fluorescence intensity of lungs by the end of treatment, and then lungs were collected for another fluorescence intensity measurement (n = 5). **(E)** The HE staining showed the metastatic area of the harvested lungs from mice with pulmonary metastasis. Bar = 500 μm. **(F)** Immunohistochemistry of the lung tissues harvested from mice with pulmonary metastasis, and the positive cells of p-STAT3, Bcl-xL, cleaved-Caspase-3 were detected. Bar = 100 μm. **(G)** Western blot assay performed on the lung tissue harvested from mice with pulmonary metastasis. Data were representative of three independent experiments. *p*-values: *p < 0.05; **p < 0.01; ***p < 0.001. ns, non-significant.

In order to investigate the combination effect of AF and ICG-001 *in vivo*, we built the tumor xenograft mice model. The results showed that combination therapy resulted in a significant reduction in tumor volume of mice compared with the vehicle and the individual treatment (**Figure 5A**). Further, at the end of the 14-day treatment, the tumors were harvested, then the IHC and western blot assay were carried on to explore the underlying mechanism. The results showed the expression level of p-STAT3 and Bcl-xL were significantly lower in the combination group than those in the individual or the control groups, and the expression level of cleaved-Caspase-3 in the combination group was significantly higher than that in the other groups (**Figures 5B, C**).

To evaluate the drug toxicity, the mice body weight, blood biochemistry, and the visceral pathology were detected. The results found that there were no statistically significant differences in the body weight among groups at the end of treatment (**Supplementary Figure S1A**); the level of the urea nitrogen in mice blood after drug treatment showed only a slight increase, but the glucose, alanine aminotransferase (ALT), aspartate transaminase (AST) levels did not significantly change (**Supplementary Figure S1B**); the pathology of lungs, livers, kidneys, spleens, and hearts of mice showed no significant change after treatment (**Supplementary Figure S1C**). These results indicated that ICG-001, AF, and combination treatment could not cause severe side effect on the experimental mice.

### Combination of AF and ICG-001 Reduces Metastasis *In Vivo*

For the purpose of exploring the effect of the AF and ICG-001 combination in suppressing colon cancer metastasis, a pulmonary metastasis mice model was built. The fluorescence intensity of lungs was detected to assess the effect of combination therapy on mice bearing with pulmonary metastasis after treated with the same treatment of the tumor xenografted mice. As the results showed, the fluorescence intensity of lungs in combination group was significantly lower than those in the individual groups and the control group (**Figure 5D**). And to confirm the combination effect on pulmonary metastasis, the lung tissue samples were harvested to perform a pathological section with HE staining. And the results indicated that the lungs in the combination group showed significant less metastatic area than those in the individual groups and the control group (**Figure 5E**). Next, the underlying mechanism was also explored by harvesting the lung tissue to perform IHC and western blot assay. The results showed the expressions of p-STAT3 and Bcl-xL were significantly downregulated, and the expression of cleaved-Caspase-3 was significantly upregulated in the combination group compared with other groups (**Figures 5F, G**). Those results indicated the mechanism of antimetastasis effect induced by combination therapy was consistent with the antitumor effect in the tumor xenograft mice model.

**Figure 6:**
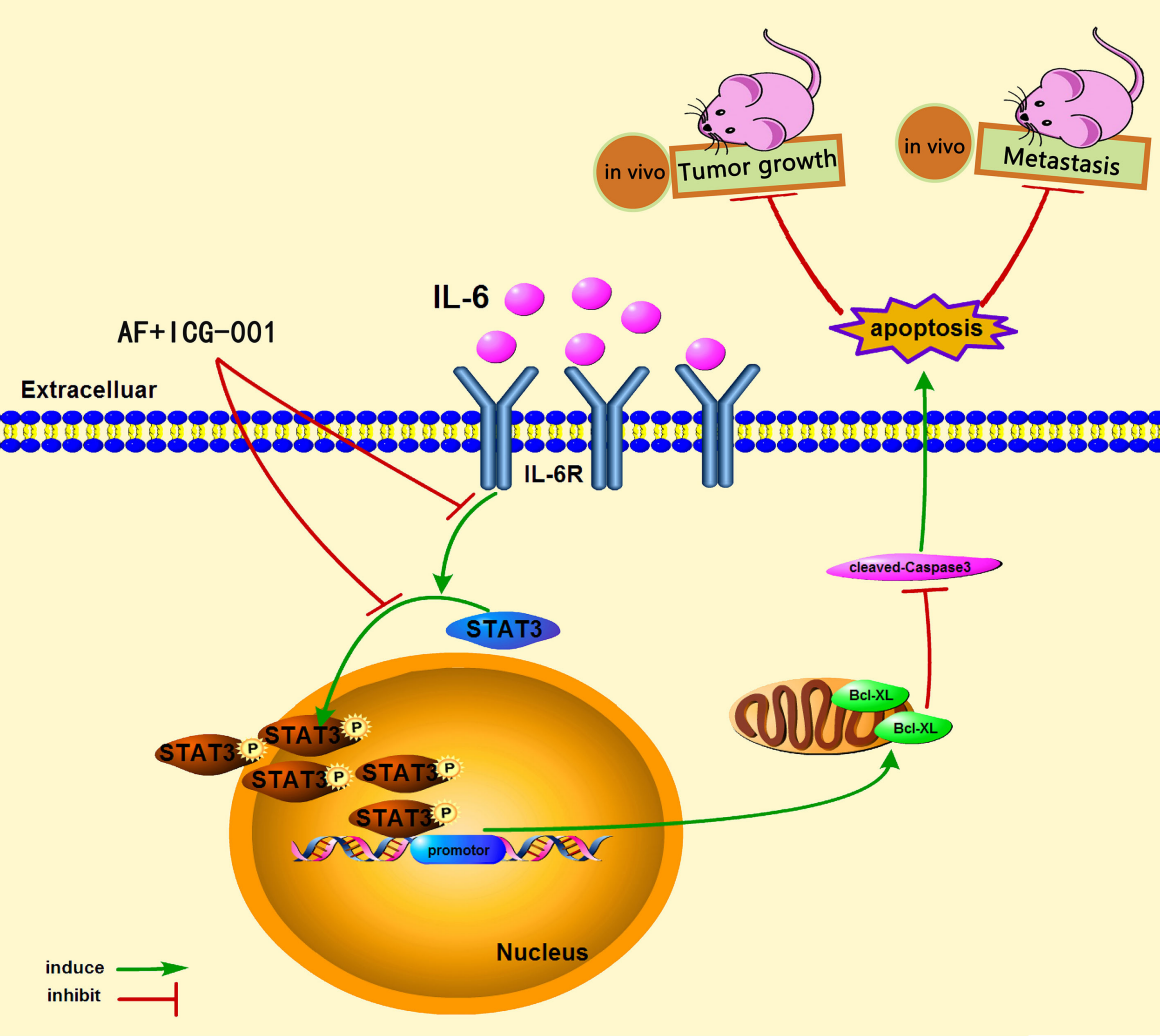
The underlying mechanism of the combination therapy of AF and ICG-001.

In conclusion, our study demonstrated that the combination therapy of AF and ICG-001 could significantly suppress the proliferation and metastasis of colon cancer *in vitro* and *in vivo via* directly inhibiting phosphorylation of STAT3. The low level of p-STAT3 could downregulate the expression of Bcl-xL, and then induce Caspased-3-dependent apoptosis of colon cancer cells (**Figure 6**).

## Discussion

Colon cancer is one of the most severe tumors all over the world, with high probability of metastasis (1, 2, 36). Drug resistance has been one of the major causes of colon cancer recurrence, and drug response appears to be an independent prognostic factor for survival (36). Therefore, the searching of more effective drugs (for example, small molecule drugs, or novel target drugs), or therapies for colon cancer is under urgent needed.

Combination therapy is applying multiple drugs for treating diseases and reducing suffering, which is usually used in colon cancer patients at various stages, especially at the advanced stage (37, 38). And some researches indicate that the combination use of 5-FU, levamisole, and leucovorin in stages II and III colon cancer patients show survival benefit (39). Other researchers also find combination therapy is effective for treating colon cancer *in vitro* and *in vivo* (40, 41). But effective combination treatment by novel targeting drugs with lower toxicity is still in exploration. Since Wnt/β-catenin signaling is critical for tumor progression and is frequently activated in colorectal cancer, researches have confirmed that small molecule inhibitors of Wnt/β-catenin pathway impaired the growth of colon cancer (42, 43); therefore, we screened the combination effect of ICG-001, a small molecule inhibitor of Wnt/β-catenin pathway, with some common chemotherapeutic drugs and novel drugs, found out the gold complex AF could coordinate well with ICG-001 to display antitumor effect in colon cancer. AF is a mature drug attested by FDA for treating human rheumatism, the safety of which has been confirmed, increasing the feasibility of transforming the present study to clinical treatment of colon cancer. It needs to be noted that in previous studies, both AF and ICG-001 show synergistical antitumor effect when combinate with other drugs (11, 13, 15, 22). And the synergistical effect is related with multiple mechanisms like ROS-related oxidative stress (13), cell apoptosis induction (22), etc. The synergistic effect of AF and ICG-001 by directly downregulating the phosphorylation of STAT3 was firstly proposed in the present study. However, we think it is also worth to further explore whether combination of AF and ICG-001 has effect on Wnt/β-catenin pathway on colon cancer cells in the future study.

The synergistical antitumor effect of AF and ICG-001 was presented on all the three colon cancer cell lines; however, we noted the different sensitivity to ICG-001 and different shape of the combination therapy dose effect diagram among those cell lines (**Supplementary Table S1** and **Figure 1B**). We guessed this might due to the genetic differences among those cell lines, and further study was required. Meanwhile, the IC_50_ of ICG-001 on colon cancer cells in several reports is lower than that in the present study (17, 18, 44, 45), and the IC_50_ of AF treatment in some reports is also lower than that in this study (5, 6, 46). We considered it might be because of some cell operation (for example, transfected with promoter), and different cell lines, cell density, drug treatment time, detection methods, etc.

The colon cancer stem cells (CCSCs) are considered as the main triggering factor of cancer progression, recurrence, and metastasis (47). The most drug resistance subsets of cancer cells with high proliferation capacity are CCSCs with self-renewal and multi-differentiation capacities (47). Exploring effective therapies targeting the CCSCs is of great significance in the treatment of colon cancer. In the present study, AF and ICG-001 combination could inhibit cell spheroids growth in 3D culture model (**Figure 1D**). And it has been confirmed that tumor-derived spheroids are purposed for the enrichment of cancer stem cells (CSCs) or cells with stem cell-related characteristics (35). Therefore, this combination treatment may also have potential as a therapy for cancer stem cell. However, further researches need to be conducted to clarify this potentiality in the future study.

Aberrant STAT3 activation triggers tumor progression through oncogenic gene expression in numerous human cancers, leading to promote tumor malignancy (31, 48). The upregulation of the phosphorylation of Tyr705 in STAT3 is frequently detected in human colon cancers (25, 26) and can induce upregulation of anti-apoptotic protein such as Bcl-2, Bcl-xL, and Mcl1 expressions to prevent apoptosis of tumor cells in multiple tumors (24, 30–32). Thus, targeting STAT3 may improve tumor progression and anticancer response. The present research showed the combination therapy of AF and ICG-001 significantly suppressed the expression of phosphorylation of STAT3 and the downstream mediator Bcl-xL both *in vitro* and *in vivo* (**Figures 3**, **4**, **5C, G**). IL-6 can directly phosphorylate STAT3 by binding to its cognate cell surface receptor, forming IL-6/IL-6R/gp130 complex, leading to the activation of associated members of the JAK family of tyrosine kinases (24, 32). However, the inhibition of phosphorylation of STAT3 caused by combination treatment could not be rescued by IL-6, and STAT3 siRNA silencing could prevent the influence of combination therapy on Bcl-xL and cleaved-Caspase-3 (**Figure 4**). Therefore, the results indicated one of the underlying mechanisms of AF and ICG-001 combination therapy is directly target p-STAT3. Here we noticed that the STAT3 level of DLD-1 cells was significantly downregulated by siRNA, but the p-STAT3 level was no significant change compared with the negative control group (**Figure 4F**). We considered the reason might be the siRNA delivery efficiency of DLD-1 cells might be not as high as that on HCT-116 and SW-480 cells. However, we could also clearly identify that the Bcl-xL and cleaved-Caspase-3 expression level of DLD cells in combination therapy group after STAT3 siRNA transfection were significantly changed compared with those in NC siRNA control group.

Colon cancer is of great percentage of malignancy and metastasis. The 5-year relative survival rate for persons with colorectal cancer is 65% until year 2019, but 5-year survival declines to 12% for stage IV disease (3). So, the inhibition of cell invasion and tumor metastasis is critical for the treatment of colon cancer. In our present study, the cell invasion ability *in vitro* (**Figure 2C**) and tumor metastasis *in vivo* (**Figures 5D, E**) were both suppressed by the combination use of AF and ICG-001, highlighting the potential of this combination therapy for malignant colon cancer patients.

In conclusion, our results demonstrated that combination of AF and ICG-001 suppressed the proliferation and metastasis of colon cancer *in vitro* and *in vivo* by directly inhibiting STAT3 phosphorylation. Therefore, this combination therapy may possess potential therapeutic properties for human colon cancer. However, more further investigations are needed to ensure the clinical efficacy of this combination therapy.

## Data Availability Statement

The raw data supporting the conclusions of this article will be made available by the authors, without undue reservation.

## Ethics Statement

The animal study was reviewed and approved by Animal Ethics Committee of the China Agricultural University.

## Author Contributions

ZYL contributed to conceptualization, methodology, data curation, and original draft preparation. HZ organized the conceptualization, methodology, software, and the review and editing of the manuscript. DL contributed to conceptualization and funding acquisition. QL arranged the data of this study. YZ and NC contributed to the methodology. ZXL organized the software. DZ and JL supported in project administration. GL was the supervision. All authors contributed to the article and approved the submitted version.

## Funding

ZYL, QL, YZ, ZXL, NC, DZ, GL, JL, and DL were funded by the National Natural Science Foundation of China (No. 31572578), and the article processing charges was funded by China Agricultural University. HZ was funded by the National Natural Science Foundation of China (No. 31902336) and the Start-up Research Grant Scheme of Hainan University (NO. KYQD(ZR)1941).

## Conflict of Interest

The authors declare that the research was conducted in the absence of any commercial or financial relationships that could be construed as a potential conflict of interest.

## Publisher’s Note

All claims expressed in this article are solely those of the authors and do not necessarily represent those of their affiliated organizations, or those of the publisher, the editors and the reviewers. Any product that may be evaluated in this article, or claim that may be made by its manufacturer, is not guaranteed or endorsed by the publisher.

## Supplementary Material

Supplementary Figure 1 The drug toxicity in tumor-bearing mice after drug treatment. **(A)** The body weight of the mice with xenograft tumor and the mice with pulmonary metastasis was measured every day during the treatment. **(B)** The blood biochemistry indexes of the mice with xenograft tumor were analyzed at the end of treatment. **(C)** The pathology of lung, liver, kidney, spleen, and heart of mice with the xenograft tumor. Bar = 100 μm. *p*-values: **p* < 0.05; ***p* < 0.01; ****p* < 0.001.
