# Supplementary material for: Combination of Auranofin and ICG-001 Suppress the Proliferation and Metastasis of Colon Cancer
Source: Front Oncol. 2021 Nov 24;11:738085. doi: 10.3389/fonc.2021.738085 (PMC8651623; doi:10.3389/fonc.2021.738085)
Supplement: Supplementary file 2 [file Table_1.docx]

**Table S1**

Dose-effect relationships of single drugs and combination in colon cancer cell lines

| Cell line | Single drugs and combination | Parameters | | | CI value at | | | | DRI value at | | | |
| --- | --- | --- | --- | --- | --- | --- | --- | --- | --- | --- | --- | --- |
|  |  | Dm | m | r | ED50 | ED70 | ED90 | ED95 | ED25 | ED50 | ED75 | ED95 |
| HCT-116 | AF | 7.84941 | 0.87572 | 0.83523 |  |  |  |  | 7.7045 | 4.85003 | 3.05312 | 1.40292 |
|  | ICG-001 | 106.394 | 0.49037 | 0.9843 |  |  |  |  | 3.89643 | 6.57395 | 11.0914 | 26.7083 |
|  | AF+ICG-001 | 17.8027 | 0.63972 | 0.95182 | 0.3583 | 0.41769 | 0.57374 | 0.75024 |  |  |  |  |
| SW-480 | AF | 9.6812 | 2.0123 | 0.97156 |  |  |  |  | 1.07499 | 1.34274 | 1.67718 | 2.43702 |
|  | ICG-001 | 264.663 | 1.2045 | 0.75918 |  |  |  |  | 2.03773 | 3.67077 | 6.61254 | 17.7759 |
|  | AF+ICG-001 | 79.3102 | 3.3958 | 0.93717 | 1.01717 | 0.74747 | 0.5613 | 0.46659 |  |  |  |  |
| DLD-1 | AF | 7.74179 | 4.88415 | 0.97553 |  |  |  |  | 3.85091 | 2.12343 | 1.17088 | 0.43068 |
|  | ICG-001 | 2697.72 | 0.70073 | 0.99452 |  |  |  |  | 35.0356 | 73.9933 | 156.27 | 548.765 |
|  | AF+ICG-001 | 40.1048 | 1.33942 | 0.98707 | 0.48445 | 0.86046 | 1.5519 | 2.32375 |  |  |  |  |

Dm: median-effect dose (concentration that inhibits cell growth by 50%), m: shape of the dose effect, r: linear correlation of the median-effect plot
